# Laparoscopic and endoscopic cooperative surgery for a gastrointestinal stromal tumor

**DOI:** 10.1055/a-2532-0876

**Published:** 2025-02-18

**Authors:** Teona Ingrid Amza, Ovidiu Mihai Arășanu, Marian Forminte, Cristiana Popp, Andrei Mihai Voiosu

**Affiliations:** 1 433892 Gastroenterology, Hospital Colentina, Bucharest, Romania; 2 433892 Surgery, Hospital Colentina, Bucharest, Romania; 3 433892 Pathology, Hospital Colentina, Bucharest, Romania; 4 87267 Faculty of Medicine, University of Medicine and Pharmacy Carol Davila Bucharest, Bucharest, Romania

A 67-year-old woman presented to the outpatient clinic with new-onset epigastric pain, with an initial abdominal ultrasound revealing a probable gastric mass. At endoscopy, we observed an approximately 5-cm subepithelial gastric tumor on the lesser curvature of the stomach and performed bite-on-bite biopsy, with a resulting histopathologic diagnosis of gastric leiomyoma being made ( Fig. 1 ). We considered submucosal tunneling techniques for removal, but, owing to the large tumor size and its position, our team opted for laparoscopic and endoscopic cooperative surgery (LECS) 1 2 for local tumor resection ( Fig. 2 ; Video 1 ).

**Fig. 1:**
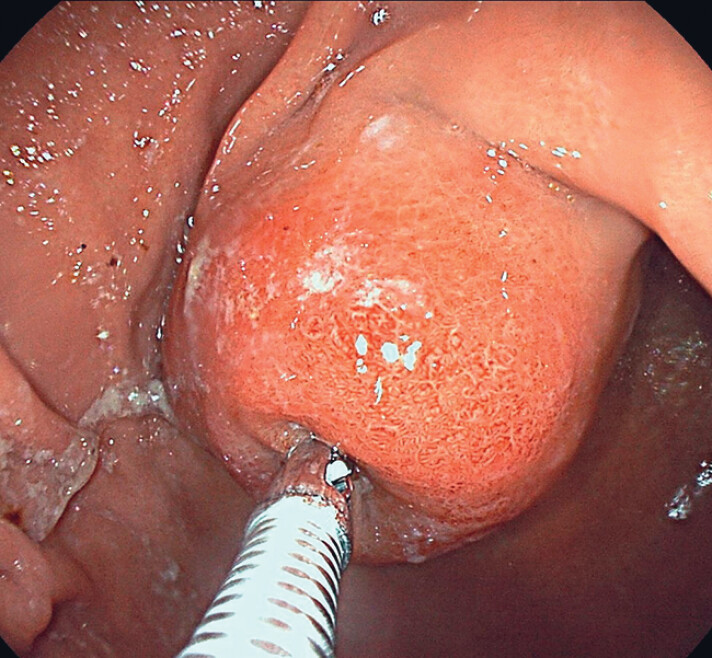
Endoscopic view showing a large subepithelial tumor protruding from the lesser curvature of the stomach.

**Fig. 2:**
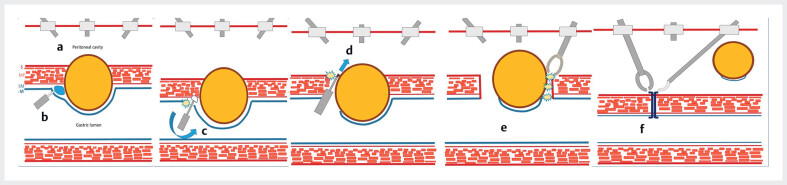
Schema showing the steps involved in the laparoscopic and endoscopic cooperative surgical procedure: **a** establishment of capnoperitoneum; **b** delineation of the tumor and formation of a submucosal cushion; **c** semicircumferential dissection of the luminal oral side; **d** controlled perforation of the gastric wall; **e** laparoscopic completion of circumferential dissection; **f** specimen retrieval, and laparoscopic suturing and closure of the wall defect.

Laparoscopic and endoscopic cooperative surgery (LECS) is performed for a large gastric tumor, with steps involving semicircumferential submucosal dissection, controlled gastric wall perforation, and laparoscopic-assisted tumor resection, followed by specimen retrieval and gastric wall defect closure.Video 1

The patient was prepared using the standard approach for laparoscopic upper gastrointestinal surgery. After capnoperitoneum had been established by the surgical team, the endoscopist marked the tumor margins and proceeded with a semicircumferential submucosal injection on the oral side and submucosal dissection with an IT2-knife. We then performed controlled perforation of the gastric wall with a needle-knife, and performed further circumferential endoscopic dissection with laparoscopic assistance ( Fig. 3 ). The surgical team flipped the tumor into the peritoneal cavity and completed the resection. The specimen was retrieved through a laparoscopic port in a protective plastic bag, and the gastric wall defect was sutured. The total procedure time was 130 minutes. The patient was discharged after 6 days, having experienced no adverse events.

**Fig. 3:**
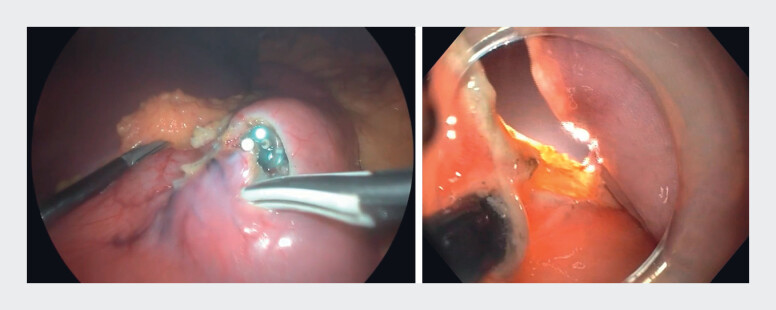
Simultaneous endoscopic and laparoscopic views during circumferential dissection.

Pathology confirmed an R0 resection of a 5-cm gastrointestinal stromal tumor (GIST) with a low mitotic index (Ki-67 of 1%) ( Fig. 4 ). In contrast to the initial diagnosis of leiomyoma, gastric GIST carries a theoretical risk of tumor seeding when resected without a true “no-touch” technique; however, because of the intact specimen and the absence of risk factors, the multidisciplinary team recommended follow-up gastroscopy and computed tomography scanning in 6 months.

**Fig. 4:**
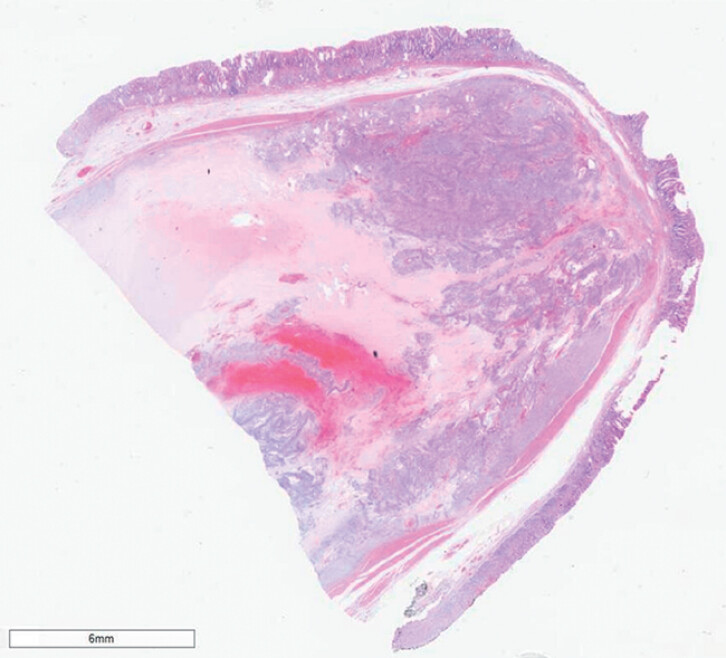
Microscopic appearance of the resected specimen showing a gastric gastrointestinal stromal tumor with clear resection margins and the typical spindle-cell architecture.

This case highlights the safety and effectiveness of underused collaborative techniques such as LECS in achieving complete tumor resection, while preserving organ function and the patient’s quality of life.

Endoscopy_UCTN_Code_TTT_1AT_2AD

## Notes

Endoscopy E-Videos https://eref.thieme.de/e-videos
*E-Videos* is an open access online section of the journal *Endoscopy* , reporting on interesting cases and new techniques in gastroenterological endoscopy. All papers include a high-quality video and are published with a Creative Commons CC-BY license. Endoscopy E-Videos qualify for HINARI discounts and waivers and eligibility is automatically checked during the submission process. We grant 100% waivers to articles whose corresponding authors are based in Group A countries and 50% waivers to those who are based in Group B countries as classified by Research4Life (see: https://www.research4life.org/access/eligibility/ ). This section has its own submission website at https://mc.manuscriptcentral.com/e-videos .
